# An Unusual Stress Fracture in an Archer with Hypophosphatasia

**DOI:** 10.1155/2013/350236

**Published:** 2013-12-09

**Authors:** Umut Yavuz, Sami Sökücü, Bilal Demir, Evren Akpınar, Osman Lapçin, Yunus Atıcı, Yavuz Kabukçuoğlu

**Affiliations:** MS Baltalimani Bone Diseases Training and Research Hospital, Orthopaedics and Traumatology Department, 34470 Istanbul, Turkey

## Abstract

We report a 45-year-old male archer with stress fracture in his left ulna on the background of adult type of hypophosphatasia. The patient presented to several medical centers for pain around the left elbow and received medical treatment upon diagnosis of tenosynovitis. History of the patient revealed that he had had diagnosis of hypophosphatasia ten years ago and underwent percutaneous screwing for stress fracture on both of his femoral necks. Upon finding nondisplaced stress fracture on proximal metaphysis of the ulna on X-ray, the patient underwent magnetic resonance imaging (MRI) in order to exclude pathological causes. No additional pathology was observed in MRI scanning. The patient's sportive activities were restricted for 6 weeks and he received conservative management with arm slings. Adult type of hypophosphatasia is a disease manifesting with widespread osteoporosis and presenting with low serum level of alkali phosphatase (ALP). Stress fracture should definitely be considered in the patients with history of hypophosphatasia and refractory extremity pain.

## 1. Introduction

Repetitive sports requiring using strength in the forearm may cause stress fractures. The risk of fracture further increases in presence of a systemic condition reducing bone quality such as hypophosphatasia [1]. Stress fractures in the upper extremity are frequently seen on the ulnar bone and on its middle 1/3 portion because of its thin cortical layer which is vulnerable to torsional forces [2]. Cases with stress fracture in the forearm have been reported in the athletes of basketball, weight lifting, baseball, or arms drill although it is usually reported to be unilateral [1–6].

We reported a male archer who had diagnosis of hypophosphatasia and presenting with stress fracture on his ulnar bone following complaint of pain around the left elbow.

## 2. Case Report

**Figure 1:**
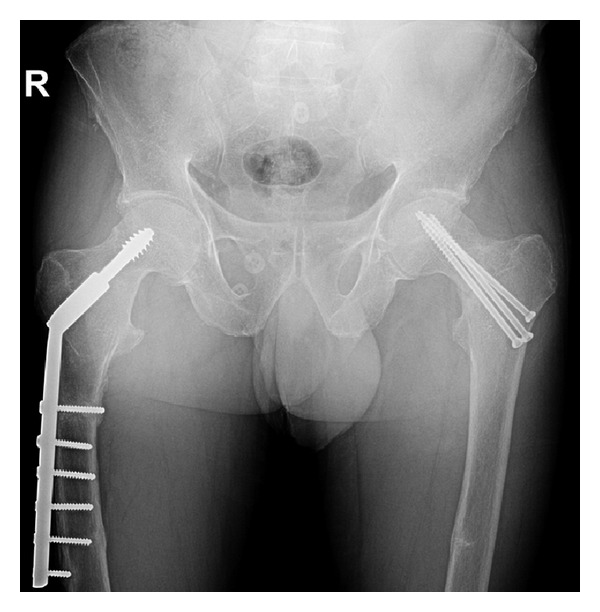
Anteroposterior X-ray showing bilateral hip fracture that underwent screwing.

A 45-year-old professional archer presented to our outpatient clinic with complaint of pain being more intense around his left elbow and radiating to the left wrist. His complaints began during training and continued after it. History of the patient revealed that he had diagnosis of hypophosphatasia ten years ago and underwent percutaneous screwing for stress fracture on both of his femoral necks (Figure 1). The patient had taken analgesics at the beginning of his complaints but presented to several hospitals upon worsening of his pain. He did not have radiological examination in those centers and had medical management with several diagnoses such as tenosynovitis, epicondylitis, and myalgia. The patient had no history of previous trauma. Except for localized pain, he had no pathological motor or sensorial examination findings to suggest neurovascular conditions.

**Figure 2:**
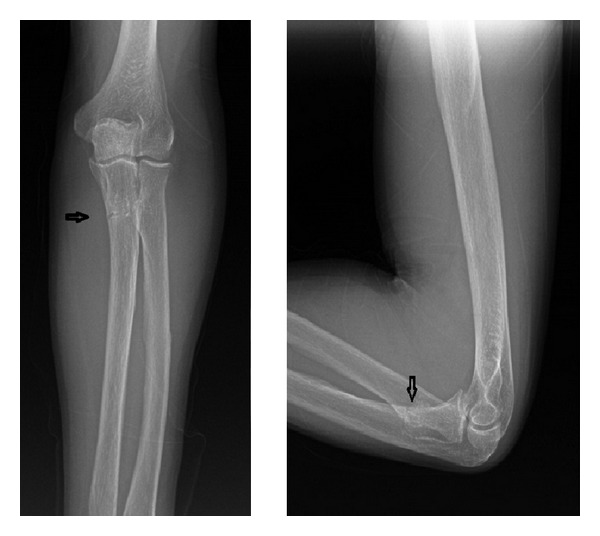
Anteroposterior and lateral X-rays showing stress fracture in proximal metaphysis of left ulna.

**Figure 3:**
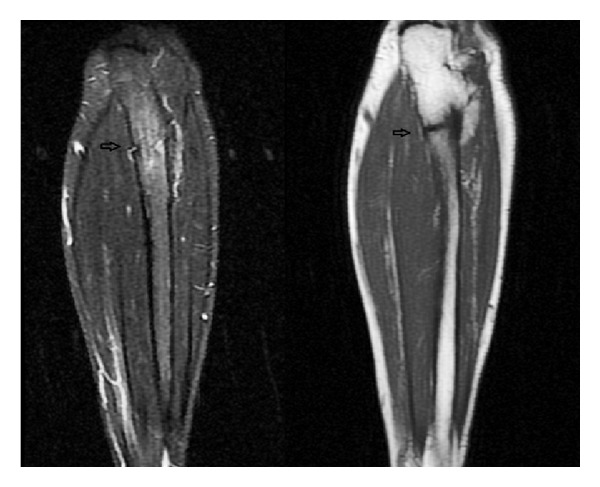
Coronal sections showing stress fracture line and surrounding soft tissue oedema in T1 and T2 sequences of the patient's forearm MRI.

On the physical examination, there was tenderness with pressing on the midproximal ulnar region. Active and passive pronation-supination movements were aggravating the pain although the range of motion (ROM) was full on the elbow. On anteroposterior and lateral radiographies, nonsegmented fracture line was seen on the proximal left ulna beginning from the medial cortex and extending to the lateral cortex (Figure 2). Stress fracture was suspected because there was no finding on the physical examination to suggest presence of infection and the patient had no history of trauma. MRI examination was performed to exclude other pathological causes although the patient had history of hypophosphatasia. On MRI scanning, there was a linear fracture line suggesting stress fracture at the level of proximal metaphysis of ulnar bone along with edema in surrounding soft tissues including medulla of the fracture (Figure 3). No additional finding was observed to suggest malignancy or other pathological conditions.

Arm sling was recommended to the patient and he was asked to suspend his sportive activities for 6 weeks. Medical treatment began for pain complaint. Radiological control films were ordered at intervals of 3 weeks. Active forced exercises began following observation of callus formation.

## 3. Discussion

Although ulnar stress fractures have been reported for several sportive branches in the previous works, we did not find forearm stress fracture developing on the background of hypophosphatasia in the literature. Additionally, fractures of the metaphysis are rare although they are usually seen on middiaphysis of the ulna [2]. We reported here a patient with hypophosphatasia who developed stress fracture on his proximal ulnar metaphysis following long trainings of archery.

Hypophosphatasia is an autosomal recessive disease. It has perinatal, infantile, juvenile, and adult forms. Adult type usually manifests in the middle of life. The first manifestation is foot pain that may develop due to stress fracture on the metatarsals. Stress fractures occurring on the diaphysis or upper end of the femur may cause pain on the thigh or hips. Frequently, lower extremity stress fractures have been observed especially on the metatarsals. Our patient had stress fracture on his upper extremity. We consider that the reason for this was that the patient was an archer and torsional force was so much on his arm [1].

Stress fracture on the ulnar bone was first described by Troell et al. in 1941 [7]. Since the original case, many cases with stress fracture of ulna have been reported in several sportive branches such as tennis, weight lifting, baseball, and polo [8–12].

Stress fractures on the nonweight bearing bones may be easily confused with pathological fractures if the clinical course of the patient is not obvious and the patient has a history of malignancy in another site. Thus for these patients, history should be taken carefully and detailed physical examination should be done. Additionally, MRI scanning should be performed in the area and scintigraphic scanning may be done in suspicious cases. Medical conditions that may cause the condition should definitely be excluded. When the patient has been seen in the clinic, assays of calcium, phosphorus, parathyroid hormone, vitamin D, and bone densitometry may be helpful for investigating metabolic conditions. Endocrinology consultation must be absolutely performed even though no abnormality is found in the investigations. Laboratory investigations and required consultations were made because of history and age of our patient.

Since there was no pathological or metabolic problem, the possible mechanism was considered to be a combination of excessive axial force occurring during forced extension and de-rotational force occurring to avoid release of the arch [3–5]. No pathological lesion (soft tissue or bone tumor, periosteal reaction, etc.) was observed that might cause fracture on MRI scanning and the fracture was progressing in the process of healing without complication.

In conclusion, diagnosis of stress fracture should absolutely be considered for pain on the forearm or around the elbow in the athletes excessively using their arms and frequently exposing to them tractional-rotational forces. However, risk of fracture further increases in the presence of systemic conditions reducing quality of bone such as hypophosphatasia. Therefore, carefully querying history of the patient may enable early initiation of the treatment as well as avoid serious disabilities that may develop.
